# Gemi: PCR Primers Prediction from Multiple Alignments

**DOI:** 10.1155/2012/783138

**Published:** 2012-12-19

**Authors:** Haitham Sobhy, Philippe Colson

**Affiliations:** ^1^Facultés de Médecine et de Pharmacie, Aix Marseille Université, URMITE, UM 63, CNRS 7278, INSERM, U1095, 13385 Marseille Cedex 05, France; ^2^Pôle des Maladies Infectieuses et Tropicales Clinique et Biologique, Fédération de Bactériologie-Hygiène-Virologie, IHU Méditerranée Infection, Centre Hospitalier-Universitaire Timone, Assistance Publique-Hôpitaux de Marseille, 13385 Marseille Cedex 05, France

## Abstract

Designing primers and probes for polymerase chain reaction (PCR) is a preliminary and critical step that requires the identification of highly conserved regions in a given set of sequences. This task can be challenging if the targeted sequences display a high level of diversity, as frequently encountered in microbiologic studies. We developed Gemi, an automated, fast, and easy-to-use bioinformatics tool with a user-friendly interface to design primers and probes based on multiple aligned sequences. This tool can be used for the purpose of real-time and conventional PCR and can deal efficiently with large sets of sequences of a large size.

## 1. Introduction

Polymerase chain reaction (PCR) has been increasingly used over the last two decades to detect, quantify, and/or sequence nucleic acids from various sources [1, 2]. The number of publications in PubMed referencing “PCR” has increased from 2846 in 1990 to 20,426 in 2000 and 44,231 in 2010. This method has a wide range of applications, particularly in the field of microbiology [3–5] where primers and probes are often designed with the aim to hybridize to the greatest number of genome sequences for given groups of viruses, bacteria, or parasites [5–7]. However, designs can be challenging if primers and probes are meant to hybridize to sequences with considerable nucleotide diversity; the task becomes more complicated as the nucleotide diversity increases. Thus, identifying conserved regions in the targeted nucleotide sequences is a critical step in PCR primer design [5–9].

Several tools are available to design primers [6–14]. However, these tools often present limitations in their capabilities to parse numerous and/or large sequences, which are frequently encountered situations, or to deal with degenerate positions, and some of them are not easily usable without skill in bioinformatics.

Here, we present Gemi, which means “to find” in ancient Egyptian, a simple, automated, fast, and versatile tool to find universal primers and probes within a set of multiple, variable, and long sequences. The main criterion used to identify primers and probes in Gemi is nucleotide conservation, but our tool provides the dissociation temperature (*T*_*d*_), length, and GC percentage in the final output file for each of the chosen primers or probes. The application executes directly on a PC computer and provides a simple and user-friendly interface that allows designing primers easily and quickly. In addition, Gemi can parse several hundred long (>1 kilobase) sequences within seconds. We believe that our tool can be particularly useful in the field of microbiology.

## 2. Algorithm and Method

The input file for Gemi is a multiple aligned FASTA file. Once it is uploaded to the program, a consensus sequence will be constructed. Gemi also accepts a single sequence (that can be manually curated) and uses it as a consensus. Unlike other programs, degenerate nucleotides are included in the consensus sequence and follow the IUPAC-IUB nomenclature system [15] (see Section 1 in the supplementary file, SI-1 of the Supplementary Material available online at doi:10.1155/2012/783138). Gemi then searches for primers and probes by sliding a window of a chosen size, which corresponds to the required size of the PCR product, along the full-length consensus sequence. The step value by which the window slides is the sliding value (Sections 2 and 3 in the supplementary file, SI-1). 

**Figure 1:**
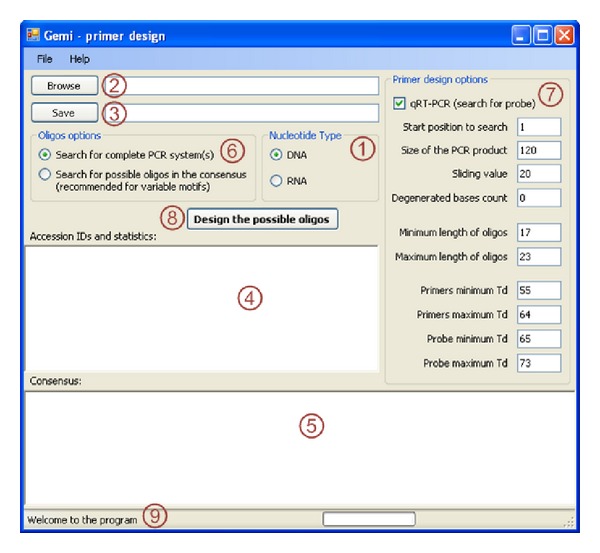
Screenshot of the main window of Gemi. (1) Switch from DNA to RNA, (2) browse for the input FASTA file, (3) save the output tabulated text file, (4) the accession numbers and the percentage of the conservation in the consensus will appear in this area, (5) the consensus sequence is written in this area, (6) switch between the options, (7) choose to design probes by ticking real-time PCR, the parameters can be edited in this menu, (8) finally, click to design the primers, and (9) the program progress will be seen in the status bar.

**Figure 2:**
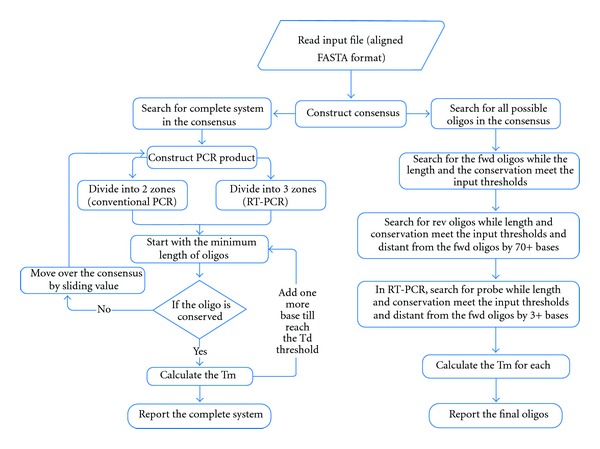
Flow chart explaining the procedure used by Gemi to find oligos. The first step is to load the sequences to Gemi. Then, the tool parses them and searches for the conserved regions and reports them in the final file. fwd, forward; rev, reverse; and RT-PCR, real-time PCR.

Several parameters can be modified by the user from the main window, although default values are provided for each parameter (Figure 1). These parameters include the size of the sliding window, the sliding value, the number of degenerate positions, and the size and *T*_*d*_ of the oligos (Figure 2). The default sliding value is 20. Another default value proposed for the identification of appropriate oligos is that the number of variable sites is zero at the 3′ end positions of the primers and probes. Another criterion is that the appropriate oligos must not contain more than three variable/degenerate nucleotide positions. However, more relaxed parameters can be chosen. 

Two options have been implemented in Gemi to identify potential oligos, which can be chosen by ticking boxes (Figure 1). The first option consists of delineating a size for the nucleotide fragment that will contain hybridization sites for the primers and probes. One possibility is to choose a short window size to design real-time PCR primer/probe sets, classically, <150 nucleotides. This possibility requires ticking the “search for probe” box. Another possibility is to choose a larger window size to design primers for Sanger sequencing, classically, >200 nucleotides. A second option consists of using Gemi without delineating a size for the nucleotide fragment that will contain hybridization sites for the primers and probes. This option will result in the generation of a list of all possible oligos along the consensus sequence, whatever their respective location, with the start and end positions of the oligos reported. This latter option is particularly convenient for identifying primers in highly variable sequences, when first options failed, and it allows the user to manually select the best combination of proposed oligos (Figure 2 and Section 3 in the supplementary information, SI-1).

Regardless of the chosen option, the final report presents the sequence, length, GC content, *T*_*d*_, and position on the consensus sequence for each oligo. The *T*_*d*_ of small oligos is estimated using the Wallace rule for the dissociation temperature [16, 17]. For longer oligos, the nearest-neighbor method is used to calculate the melting temperature (*T*_*m*_) [18]. Here, the *T*_*d*_ is calculated using the equation in [17]: *T*_*d*_ = 2°C ∗ (#A + #T) + 4°C ∗ (#C + #G), where “#” refers to the number of As, Cs, Gs, or Ts in the oligo. 

## 3. Results and Discussion

We developed Gemi to supply the critical needs for the design of PCR primers and probes with an easy-to-use, fast and efficient. Several other tools for the design of PCR systems have been previously described [10]. Nonetheless, some limitations can be pointed out for these tools [19–33]. The first limitation is that some tools, such as Primer3 that is an online and powerful tool to design primers based on a single, short and conserved sequence, cannot parse sequences with degenerate bases, what can be accomplished by Gemi [19]. Other software as BatchPrimer3 or Primaclade accepts only one sequence [21, 22]. Other tools exist that can overcome this shortcoming [29–33], such as PrimerIdent, which accepts only eight sequences, one of them being used as template [29]. GeneFisher can parse multiple sequences but fails to deal with sequences with degenerate bases [30]. The web-based tool Greene SCPrimer designs degenerate primers from multiple sequence alignments by constructing phylogenetic tree, which is a slow process [32]. The easyPAC tool can design degenerate primers and also performs mapping to reference files for real-time PCR, but it performs slower than Gemi for the primer and probe design [33]. We previously described SVARAP for the analysis of sequence variability and primer design [6], which can analyze a maximum of 100 sequences with a maximal length of 4,000 nucleotides. Other tools have specific applications, such as PhiSiGns that identifies gene signatures in phage genomes [34]. Besides, some tools for the design of PCR systems require bioinformatics skills, such as “Prosig” [35] or the PriMux package that is based on python scripts to search for primers and probes on nonaligned multiple sequences [7]. 

Existing tools often search for oligos by taking into account parameters such as the GC-content, the *T*_*m*_, or the formation of secondary structures. However, the most critical issue for several PCR-based assays is the identification of conserved regions where primers and probes can hybridize, in addition to the length and number of query sequences. These issues are particularly important in the field of microbiology. Moreover, the lack of user-friendly interface and cross-platform tool are challenging issues for biologist without prior knowledge of the programming tools. 

Gemi has several advantages compared to other tools (Table 1). It is able to automatically and rapidly predict PCR primers for numerous long and variable sequences. Additionally, Gemi can be used to design PCR systems for both real-time PCR and sequencing. Moreover, no training in bioinformatics is required to use Gemi, which has a user-friendly interface.

Using Gemi on a PC with 512 MB RAM, it succeeded to construct consensus and to identify primers and probes among 61 aligned full-length hepatitis C virus genomes with a length of about 10000 nucleotides within few seconds (Table 1 and Section 4 in supplementary file, SI-1), while easyPAC failed to identify any primer and Greene SCPrimer hardly runs to design primers even with shorter sequences. 

Some advanced options such as identification of secondary structures, and prediction of hairpins and primer-dimer formation are not presented in this version; these issues may be addressed in future versions of Gemi. Alternatively, prediction of the hairpin or dimer formation could be determined by other tools as OligoCalc tool [36]. 

## 4. Availability and Implementation

Gemi is a cross-platform application which is distributed under GNU-GPL license and is free to use for academic and research purposes. The portable desktop version of this tool facilitates its free distribution and usage. The software and documentation are freely available for research use at https://sourceforge.net/projects/gemi/. A script version of Gemi will be available upon request.

The tool runs on Windows 7 without any preliminary installations. For older versions, the software requires Microsoft.NET (Dot Net) Framework version 2.0, which is freely available from Microsoft website. For the Linux, Ubuntu, and Mac OS X users, please download Mono tool to run the software from http://www.mono-project.com/ or http://monodevelop.com/ (see the program's user guide).

The input file is a standard FASTA format file that contains a single sequence or a multiple sequence alignment, which can be created using any available alignment tool. The output file is generated as a tabulated text file that is easily read using any text processing program and contains the sequence of PCR product (if chosen), sequence of primers and probes (if chosen), positions of the oligos on the consensus, *T*_*d*_, number of degenerate nucleotide, and GC content (see supplementary file and program's user guide). 

## 5. Conclusions

We presented a simple, robust and fast tool, GEMI, which fulfils the regular requirements for biologists to design primers and probes. We believe that this tool can be helpful for research or diagnosis for a wide range of applications that includes detection, quantification, and genotyping in microbiology.

## Supplementary Material

Supplemental data on methods and performance.

## Figures and Tables

**Table 1:** Comparison between Gemi and other existing public tools.

Criteria	Gemi	Primer3	easyPAC
Simplicity	Yes	Yes	Yes
Fast^1^	Yes	Yes	∗
User friendly	Yes	Yes	Yes
Multiple and divergent sequences^2^	Yes	NA	#
Long sequences^3^	Yes	NA	∗
Cross-platform	Yes	Online	Yes
Probes' design	Yes	Yes	NA
GC content	Yes	Yes	Yes
Temperature	Td	Tm	Tm
Temperature range^4^	Yes	NA	#
Hairpin structure	NA	Yes	#
Parameters^5^	Basic	Advanced	Advanced

The table represents a comparison between Gemi, Primer3, and easyPAC tools. (Yes) denotes it is covered by the tool, (NA) means not offered by the tool, (∗) means offered but Gemi performs better in this function, while (#) means this option is offered referring to the paper.

^1^Gemi can retrieve primers and probes within seconds (Section  4 in supplementary document, SI-1). Primer3 searches for primers within short fragment of the sequence; its performance is relatively fast. EasyPAC performed slower than Gemi.

^2^Gemi succeeded to find primers and probes for multiple and divergent (aligned) sequences with about 30% identity, while Primer3 could not parse divergent sequences and easyPAC failed to retrieve any primer.

^3^Gemi successfully presented primers and probes for input sequences of about 10 kbp; the same process cannot be accomplished by Primer3 and easyPAC.

^4^In case of degenerate nucleotides in a position within primers, Gemi reports the temperature range of these nucleotides. Based on the paper, easyPAC reports it as well.

^5^Although Primer3 and easyPAC tools offer advanced functions, Gemi is designed to cover the basic needs of biomedical field to find reliable primers within minutes with user-friendly interface.
