# Supplementary material for: Gemi: PCR Primers Prediction from Multiple Alignments
Source: Comp Funct Genomics. 2012 Dec 19;2012:783138. doi: 10.1155/2012/783138 (PMC3535827; doi:10.1155/2012/783138)

Gemi: PCR primers prediction from multiple alignments

Supplementary file

Section 1. The degenerated bases and the representing IUPAC symbols

Table 1 | IUPAC-IUB nomenclatures

List of the degenerated bases and their IUPAC symbols representation, the third column contains the complementary bases used by the Gemi tool*.

Symbol	Meaning	Base represented	Complement
	**Only one conserved base**		
**A**	Adenine	A	T or U
**C**	Cytosine	C	G
**G**	Guanine	G	C
**T or U**	Thymine (DNA) or Uracil (RNA)	T or U (RNA)	A
	**2 degenerated bases**		
**R**	puRine	A or G	Y
**Y**	pYrimidine	C or T	R
**W**	Weak	A or T	W
**S**	Strong	C or G	S
**M**	aMino	A or C	K
**K**	Keto	G or T	M
	**3 degenerated bases**		
**V**	not T or U (RNA), V comes after T	A, C or G	B
**H**	not G, H comes after G	A, C or T	D
**D**	not C, D comes after C	A, G or T	H
**B**	not A, B comes after A	C, G or T	V
	**4 degenerated bases**		
**N or X**	Unknown or aNy	A, C, G or T	N

*** References:**

http://arep.med.harvard.edu/labgc/adnan/projects/Utilities/revcomp.html

http://www.ebi.ac.uk/2can/tutorials/aa.html

<u>Section 2</u>. Building the consensus

Gemi tool runs pairwise comparison between the nucleotides harboured in the same position on different sequences. This step ensures that all the nucleotides exist on a given position are represented on the consensus sequence, regardless of their prevalence or abundance on the sequence. After comparing the nucleotides, if the nucleotides are identical in all sequences, Gemi appends the nucleotide to the consensus. In contrast, if different nucleotides are harboured at a position in the multiple alignments, the tool will incorporate the corresponding IUPAC symbol (degenerate base) into the consensus sequences (table 2). Here, the degenerate base means not A, C, G nor T. In case of a gap, the tool represents a gap at this position.
For the standard bases tool calculates the Td by the simplest formula mentioned above. In case of degenerate base, the tool calculates it as maximum and minimum Td based on the represented standard bases (table 2).

Table 2 | Representation of the method used to build the consensus

	1	2	3	4	5	6	7	8	9
**Gene 1**	A	C	G	A	C	T	A	T	T
**Gene 2**	A	C	G	A	-	C	W	T	A
**Gene 3**	A	T	G	G	C	G	T	S	C
**Gene 4**	A	C	C	A	C	G	T	T	G
**Consensus**	A	M	S	R	-	B	W	B	N

Each number represents a nucleotide position on sequences and consensus; underneath the nucleotides are presented. By comparing the nucleotides against each others the final consensus is built-up. Position (1) contains only 'A' bases therefore, it is written in consensus sequence. The (2) contains C and T bases, results in M. If one of the sequences contains a gap like in (5), the final position will be gap as well. The (7) combines between A, T and W (A or T), the final is W. Position (8) harbour T and S (C or G); the final is B, which represents C, G or T bases. If the position harbours all the bases like in (9), N symbol will be written to the consensus sequence. In case of M symbol, which represents C or T bases, the tool calculates Td for M once as T (multiply by 2°C) and then as C (multiply by 4°C) and the minimum Td and maximum Td are reported.

Note on building the consensus sequence

This initial version does not generate any graphical representation of the variability or relative abundance of nucleotides at each position along the sequence alignment.
In some cases, the generation of degenerate bases in the consensus sequence, regardless of the number of sequences that does not harbour the majority nucleotide in the alignment. However, Gemi can accept a manually curated consensus sequence in such cases.
An example, each degenerated base mixes between the A, C, G or T bases. The percentage of each base of them the degenerated position is called variability, which is useful for calculating the concentration of each base on the primers. Therefore, the degenerated bases will be written to consensus even if the variability is low.
For example, if there is 10 sequences, 9 of them are A while only one is T, the final position in the consensus is W (which means A or T). The tool does not report the percentage of any of these nucleotides (neither A nor T). In this case, the tool tends to misrepresent the value of each base within this position.
In the current version, Gemi does not report the position variability or the percentage of each base in the specified position. This issue will be noticed in the future version. Alternatively, if a single sequence is provided the tool will deal with it as consensus sequence and predict the possible oligos within it.

Section 3. Algorithm and method

Once the input file (the multiple alignments in FASTA format) has been loaded into the program, the consensus sequence will be built. Gemi accepts the degenerate nucleotides (non A, C, G or T). Figure 1 in the manuscript shows the work flow of the process by which Gemi finds the primers; while, figure 3 shows the pseudo-code illustrating this procedure.

There are two options to search for oligos with the Gemi tool:

1. The First option is used to mine for full system, including PCR product with forward and reverse primers, and probe in case of real-time PCR. The user can control the length of the PCR product, the length of the oligos and their dissociation temperatures (Td) from the main window by editing the values on the specified text area. The final output file contains the full sequence of the PCR products on one hand, and the sequences of the primers and probes, their length, their Td and the number of degenerate bases on each of the oligos. Then, the tool moves along the consensus and search for other PCR product, as illustrated on figure 1.

Figure 1 | Schematic diagram of the searching PCR system step

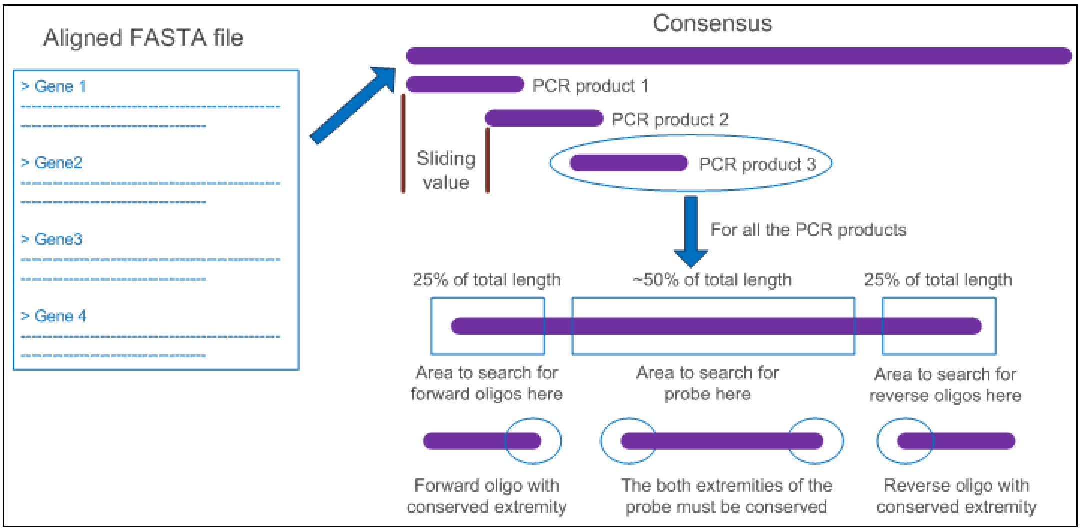

2. The second option is to find all the oligos in the consensus sequence. Here, the tool does not construct PCR systems. Instead, it deals with the whole consensus as a PCR product and search on it for oligos. Then, the user can choose any of the proposed oligos.

Firstly, the tool searches for all possible forward primers. The search process starts from the start position entered by the user on the main screen. Then, Gemi moves along the consensus sequence from a number of nucleotides corresponding to the sliding value, and it searches for new primers until the end of the consensus.

After the tool has finished mining for forward primers, it searches for reverse, then for probe if needed, using the same strategy that it has been mentioned above (figure 2).

Figure 2 | Schematic diagram of the searching list of all oligos

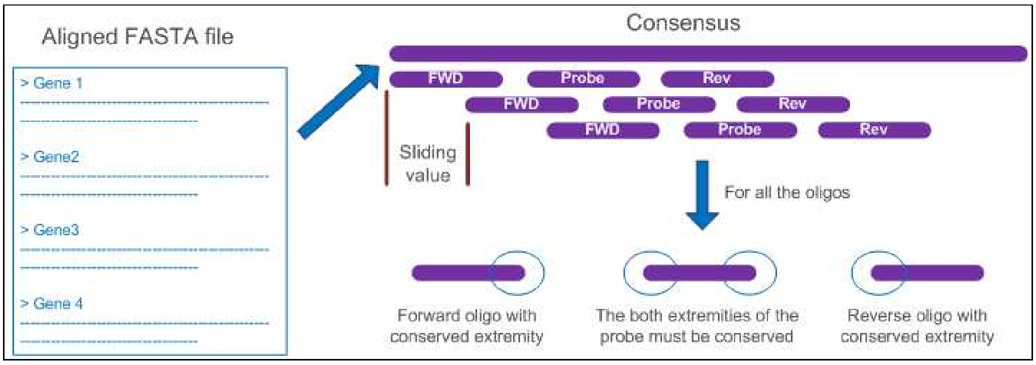

The system moves over the consensus by distance equivalent to the sliding value entered by user on the main window. Therefore, each two searches are separated from each others by this sliding value or distance. For fussy search, it is recommended to use small sliding value. Small values are also recommended for sequences with a high variability.

The default criteria to choose primers and/or probes are containing three conserved bases at 3' end of the fwd, at 5' end of the rev and at both extremities in case of the probe.

On the other hand, the oligo usually contains no more than 3 degenerated bases. The user can determine the number of the degenerated bases in each oligo on the main window.

The temperature calculation obeys the equation: Td = 2 * (#A+#T) + 4 * (#C+#G), where, "#" refers to the number of As, Cs, Gs or Ts in the oligo. In case of degenerated bases, the tool calculates the minimum Td and maximum Td. If the bases harbored at a same position in the multiple alignment are complementary (A/T or C/G), the minimum and maximum Td are the same. Otherwise, the tool calculates the Td for A/T by multiplying the base by 2°C and reports it as minimum Td, whereas the maximum Td refers to C/G and is multiplied by 4°C.

Figure 3 | Pseudo-code illustrating the procedure

```
Get the input FASTA file
Build up consensus

Case 1: Find a complete system
  Build PCR product with length equal to the value determined by user
    Search for the primers at extremities
    IF RT-PCR
      Search for the probe in the middle
    FOR EACH oligo start from the minimum length threshold
      IF the oligos in not variable (no IUPAC or less than the user input)
        Calculate Td
          IF the Td is more than the minimum threshold
            Mark as valid oligo
          ELSE IF less than the threshold
            Continue adding new bases till reach the maximum length
  Add the sliding distance and build new product

Case 2: Find all the oligos in the consensus
### Start of STEP_1
FOR the forward oligo equal the minimum length entered by user
  IF the oligos in not variable
    Calculate Td
    IF the Td is more than the minimum threshold
      Mark as valid oligo
    ELSE IF less than the threshold
      Continue adding new bases at the end till reach the maximum length
Add the sliding distance and build new oligo
Repeat the STEP_1 till the end of the consensus
### End of STEP_1

Repeat STEP_1 for reverse oligo
WHERE reverse is distant from the forward

IF RT-PCR
  Repeat STEP_1 for probe
  WHERE probe is distant from the forward

Report the found oligos into output file
```

Section 4. Performance and comparing with other tools

Gemi is a simple and fast tool; On PC with 512 RAM, Gemi succeeded to find primers for 61 hepatitis C virus (HCV) sequences with a length of 9769 nucleotides in seconds (the speed of the tool may differ from PC to another due to its specifications, the memory usage, etc). However, the sequences are diversified and show only 31% homology, it took 2 seconds to build the consensus (figure 4), while the prediction steps (the two options) was almost the same (figure 5 and 6). After eight minutes, easyPAC (1) failed to indentify any primer on the sequences (figure 7). A smaller sequence has been uploaded to Greene SCPrimer (2), but the tool failed to predict any primer, particularly with sequences contain gaps or degenerate symbols.

References

1. Rosenkranz D., easyPAC: A tool for fast prediction, testing and reference mapping of degenerate PCR primers from alignments or consensus sequences, Evolutionary Bioinformatics Online, 2012, 8, 151.
2. Jabado O.J., Palacios G., et al., Greene SCPrimer: a rapid comprehensive tool for designing degenerate primers from multiple sequence alignments. Nucleic Acids Res., 2006, 34, 6605-6611.

Figure 4 | Building the consensus for HCV sequences

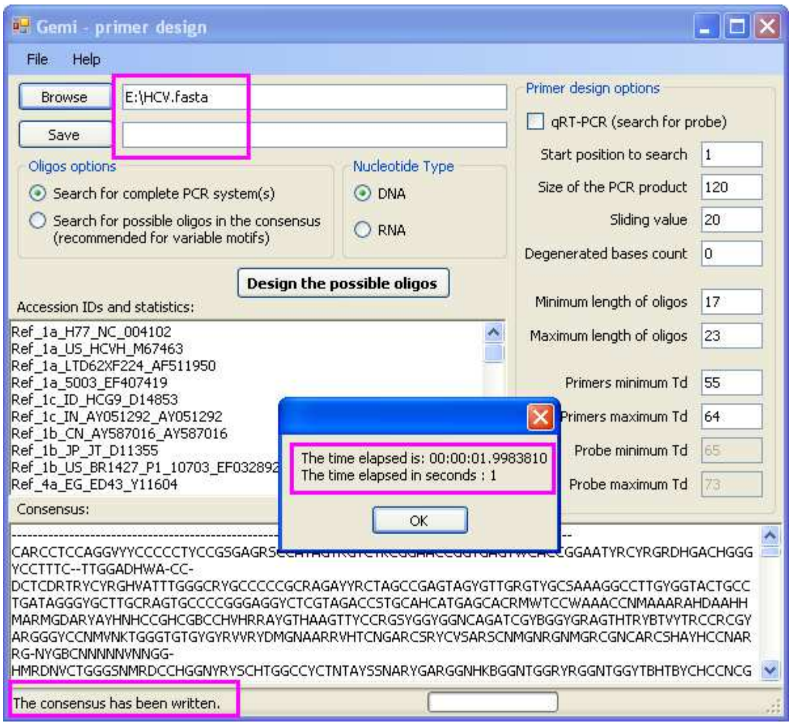

Figure 5 | Finding primers and probes using the first option

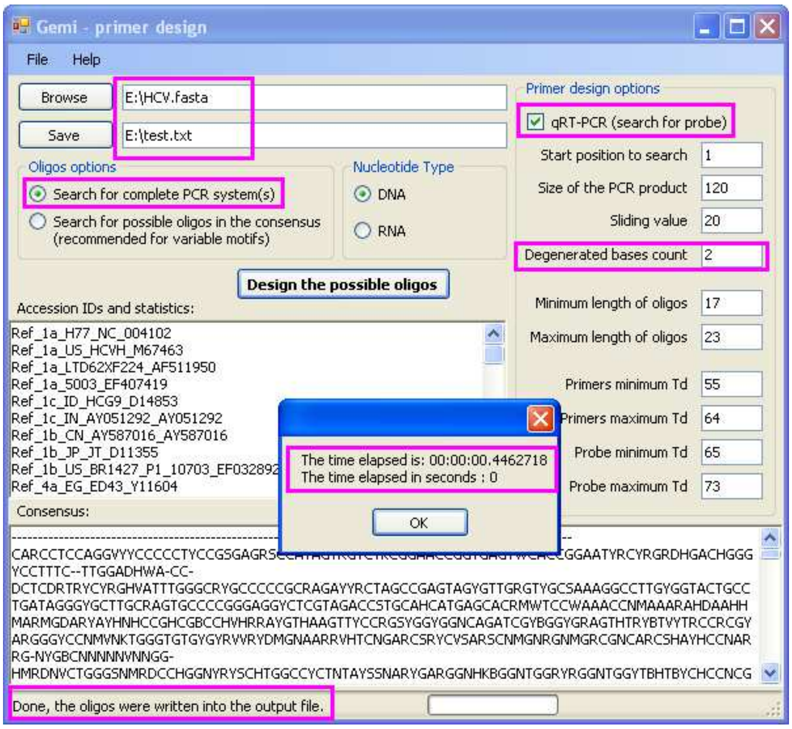

Figure 6 | Finding primers and probes using the second option

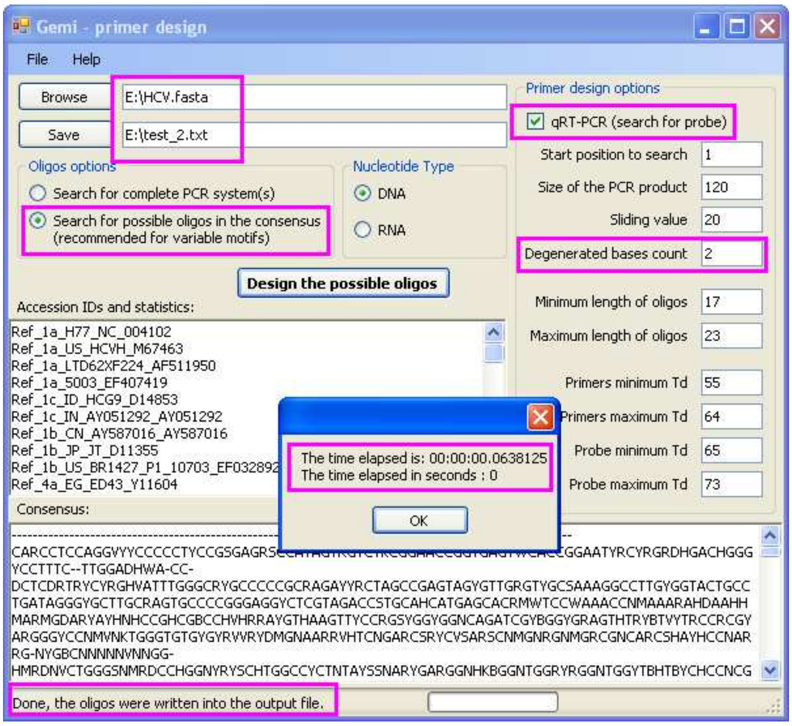

Supplement: Supplementary file 1 — Supplemental data on methods and performance. [file 783138.f1.pdf]
